# Total Parenteral Nutrition—A Team for Central Venous Line Insertion at the Bristol Royal Infirmary; Results of a Pilot Scheme

**Published:** 1984-01

**Authors:** Arthur Allan, William Sellers, Neil Mortensen, David Leaper

**Affiliations:** University Department of Surgery, Bristol Royal Infirmary, Bristol BS2 8HW; University Department of Surgery, Bristol Royal Infirmary, Bristol BS2 8HW; University Department of Surgery, Bristol Royal Infirmary, Bristol BS2 8HW; University Department of Surgery, Bristol Royal Infirmary, Bristol BS2 8HW


### Total Parenteral Nutrition—a Team for Central Venous Line Insertion at the Bristol Royal Infirmary: Results of a Pilot Scheme

**Arthur Allan M.D. F.R.C.S.**
Surgical Registrar
**William Sellers F.F.A.R.C.S.**
Senior Anaesthetic Registrar
**Neil Mortensen M.D. F.R.C.S.**
Consultant Senior Lecturer
**David Leaper M.D. Ch.M. F.R.C.S.**
Consultant Senior Lecturer
University Department of Surgery, Bristol Royal Infirmary, Bristol BS2 8HW

### WHY DO WE NEED A SERVICE FOR TOTAL PARENTERAL NUTRITION?

The use of total parenteral nutrition (TPN) to treat seriously ill patients is now an established part of hospital practice. However, the indications for this treatment are poorly defined and sometimes controversial. Fundamental questions concerning the efficacy of TPN can only be answered by measuring nutritional parameters over the course of treatment. A supervised team approach could fulfil the dual functions of providing an efficient clinical TPN service and nominate a group of individuals able to safely insert central venous catheters and supervise their nursing care.

Over the year October 1982 to October 1983, a service was offered to insert and manage central venous lines for TPN in the Bristol Royal Infirmary. As this was a pilot scheme the number of patients referrred was small and largely confined to patients in the intensive therapy unit. It was envisaged that the service would also be available to insert central venous lines for the administration of cytotoxic chemotherapy. This report summarises the problems encountered and offers suggestions which might be useful in providing a definitive hospital service.

### ORGANISATION AND SERVICE PROVIDED

The team consisted of a surgical registrar and the anaesthetic registrar in charge of the intensive therapy unit supported by two senior lecturers in general surgery. In every case, central venous access was achieved using a silicone catheter (Nutricath; Vygon (UK) Ltd) which was inserted into a subclavian vein through a subcutaneous tunnel. All catheters were inserted under fully aseptic conditions in a main operating theatre or the intensive therapy unit. The correct position of the catheter was confirmed by chest X-ray. Sterile gloves were worn by nursing staff whilst changing infusions or dressings.

### TNP RECIPES

During the early part of the year, TPN was administered using a system of 0.5 litre bottles in combination. More recently a single bag system was used (Travenol Laboratories Ltd) containing 2.5 litres of parenteral nutrient which supplied 14 g nitrogen as amino acids and 7.56 MJ (1800 kcal) as dextrose over 24 hr. In special circumstances it was possible for the pharmacy to add or subtract components from this recipe (for example additional electrolytes or vitamins).

### OUTCOME

Over the 12-month period, 18 tunnelled central venous subclavian lines were inserted in 13 men and 5 women, mean age 58 yr (range 28–79 yr). Most were suffering from gastrointestinal disease and required TPN in their management. Only one line was used for cytotoxic chemotherapy.

The average duration of parenteral nutrition was 9 days (median 7, range 2–18 days). Seven patients (38%) died during or soon after their course of TPN, but in no case was the course of nutrition thought to be a contributory factor. The remaining 11 patients (62%) left hospital alive and well.

The patient's weight was regularly recorded in only two cases and daily nitrogen balance was not measured in any patient. The mean serum albumin in 12 patients before commencing TPN was 27 g/l (range 20–37 g/l), and 26 g/l (range 20–33 g/l) after a period for TPN of between 5 to 7 days in these patients, which is not a statistically significant difference.

### COMPLICATIONS

### (a) MECHANICAL

The incidence of mechanical complications related to insertion of the catheters was low. On one occasion it was not possible to cannulate the subclavian vein. One insertion was complicated by an initial arterial puncture which required no intervention, and one other by venous bleeding leaving 16 (84%) uncomplicated insertions. Routine chest X-ray revealed no pneumothorax but several catheters needed withdrawal from the right side of the heart for correct placement.

Mechanical complications during the course of TPN were encountered in five cases. One patient pulled his own line out, and one 'fell out'. One catheter became detached from the giving set, and two catheters split. Thirteen courses (72%) of TPN were uncomplicated by mechanical problems during administration.

### (b) INFECTIVE

In four patients, the silicone catheters were suspected of being responsible for the cause of a pyrexia. In two of these cases, culture of the catheter tip after removal resulted in no organisms being grown. From one of the other two patients, one catheter tip harboured a *Staph. viridans* found on microbiological culture although colonisation was regarded as unlikely. Despite removal of the line this patient's septicaemic condition persisted and she died. From the final patient, *Staph. albus* was cultured from the central venous catheter. The patient's pyrexia settled once his catheter was removed. The overall documented infection rate was therefore 11%.

Several patients required additional insulin to regulate hyperglycaemia, but there were no serious metabolic complications.

### DISCUSSION

A team approach for the provision of TPN and insertion of central venous lines is attractive, since it provides a open service to busy clinical departments. It allows a small group of practitioners to gain considerable experience in the placement of central venous lines, and this group, in turn, could teach other interested clinicians. Such a service should minimise the complications associated with the use of feeding lines and provide a strict protocol for the supervision of their ward management. The Oxford Parenteral Nutrition team recently reported the administration of TPN to 179 patients over a 3 year period.[1] Their team includes a nursing sister engaged full time in the supervision of TPN lines on the wards, which is a very great advantage. Although the number of patients treated by the Bristol team is small, it is likely that the number will rise, and by extrapolation from the Oxford figures it may be estimated that around 50 patients a year could be managed by a TPN team at the Bristol Royal Infirmary. In addition the group of patients requiring cytotoxic chemotherapy through a central line could also be included.

In the period studied, silicone tunnelled catheters were used exclusively as these are associated with the lowest rate of catheter sepsis, and longer catheter life compared with similar polyvinyl chloride catheters.[2] The introduction of the 2.5 litre bag system has resulted in decreased handling of TPN apparatus on the ward, saving nursing time and reducing the risk of infection. The use of a standard 24 hour TPN regimen in Oxford benefited 70% of the patients treated, and had the advantage that bulk purchases could be ordered and wastage reduced.[1]

We did not aim to record nutritional parameters in our TPN patients, and found that the clinicians primarily responsible for the patients rarely did so. Careful monitoring is important. The patient should be weighed before feeding, and then twice weekly. Serum albumin should be measured, and although not essential, simple determination of nitrogen balance should be carried out regularly.[3] The introduction of a TPN 'record card' for each patient would greatly facilitate this aspect of care. All our patients were hypoalbuminaemic when feeding was commenced but serum albumin was maintained, halting progressive fall, in those patients treated with TPN for 5 or more days.

We report a 16% mechanical complication rate on insertion of the lines (8% in the Oxford group), and a 28% mechanical complication rate during the administration of the TPN (23% in the Oxford group). The number of mechanical complications resulting from placing central venous lines should decrease as the experience of a restricted group of clinicians increases, and we recommend that new lines should be sutured in place. The sepsis rate in our catheters was 11%, compared to an overall sepsis rate of 6% in the Oxford series (which was only 1.8% when silicon tunnelled lines were used). Our poor figures can be explained by the frequent handling of lines by unsupervised staff, in the absence of a strict protocol for catheter asepsis.

What then for the future? We have argued that a team approach to TPN will minimise the complications on line insertion, and silicone tunnelled lines should be used whenever possible. The TPN team should carefully monitor nutritional parameters on the ward using a TPN 'record card' system. Nursing staff must be aware that lines are solely for the administration of TPN and that extra 'junctions' in the giving set must be avoided. Nurses should always wear sterile gloves when handling the feeding bottles with liberal use of antiseptics, such as povidone iodine, when lines are disconnected. A constant infusion pump should be available to regulate the smooth running of the system and if a catheter becomes infected it must be removed and the tip sent for microbiological examination. It is only by attention to detail that a high standard can be maintained. The Oxford group estimate an expenditure of £200,000 (at 1982 prices) on TPN for 179 patients (approximately £1000 per patient). Surely, if for no other reason, TPN must be made as efficient as possible on financial grounds alone?

### REFERENCES

1. OXFORD PARENTERAL NUTRITION TEAM (1983) Total parenteral nutrition: value of a standard feeding regimen. *Br.Med.J.* **286**, 1323–1327.
2. MITCHELL, A., ATKINS, S., ROYLE, G. T. and KETTLEWELL, M. G. W. (1982) Reduced catheter sepsis and prolonged catheter life using a tunnelled silicone rubber catheter for total parenteral nutrition. *Br.J.Surg.* **69**, 420–422.
3. LEE, H. A. and HARTLEY, T. F. (1975) A method of determining daily nitrogen requirements. *Postgr.Med.J.* **51**, 441–445.

